# Optimizing the surgical paradigm for axillary osmidrosis: a retrospective cohort study integrating tumescent suction-curettage and direct-vision trimming

**DOI:** 10.3389/fsurg.2026.1946322

**Published:** 2026-09-17

**Authors:** Dan Zhang, Baoling Liang, Wei Zhang

**Affiliations:** 1 Department of Cosmetology Plastic Surgery, The First Hospital of Jilin University, Changchun, Jilin, China; 2 Department of Breast Surgery, General Surgery Center, The First Hospital of Jilin University, Changchun, Jilin, China

**Keywords:** apocrine glands, axillary osmidrosis, minimally invasive surgery, subdermal vascular plexus, suction-curettage

## Abstract

**Background:**

Axillary osmidrosis (AO) is a distressing dermatological condition driven by a complex genetic and microbiological axis. While surgical extirpation of the hyperplastic apocrine glands remains a highly effective treatment, surgeons persistently face an anatomical dilemma: achieving extensive clearance often destroys the subdermal vascular plexus, leading to severe ischemia and scarring. This study comprehensively reviews the biomolecular etiology of AO and evaluates an optimized hybrid paradigm designed to effectively address this surgical dilemma.

**Methods:**

A retrospective cohort study included 86 patients with bilateral AO, comparing the modified technique by tumescent suction-curettage combined with direct-vision trimming with traditional small-incision resection. To elucidate the tissue-preserving biomechanics, the operative steps are illustrated utilizing a representative severe case (24-year-old female, Park Grade III). Efficacy, complications, and patient satisfaction at 6 months postoperatively were analyzed.

**Results:**

The total effective rate was 97.67% in the modified group and 95.35% in the traditional group, demonstrating no statistically significant difference in clinical efficacy (*P* = 1.000). However, the modified cohort demonstrated significantly accelerated wound healing, minimal intraoperative bleeding, and lower complication rates (*P* < 0.05). Overall patient satisfaction was significantly higher in the modified group (93.02% vs. 72.09%, *P* < 0.05), benefiting from highly concealed scarring and the absence of flap necrosis.

**Conclusion:**

By synergizing atraumatic negative-pressure defatting to safeguard microcirculation with direct-vision trimming to eliminate surgical blind spots, the modified technique achieves comparable efficacy to traditional surgery while minimizing morbidities. It translates fundamental biomolecular mechanisms into a highly reproducible clinical surgical option.

## 1 Introduction

Axillary osmidrosis (AO), commonly known as bromhidrosis, is a distressing dermatological condition characterized by an offensive, pungent malodor originating from the axillae (1–3). Although devoid of systemic physiological threat, AO inflicts profound psychosocial morbidity, severely impairing patients’ daily social interactions, emotional well-being, and professional trajectories (4–6).

### 1.1 The biomolecular and microbiological pathogenesis of AO

To comprehend the strict necessity of precise surgical intervention, a profound understanding of the disease's basic biomolecular pathogenesis is requisite. The etiology of AO is governed by a complex, multi-tiered “Genetic-Microbiome-Endocrine Axis.”

At the genetic and histological levels, AO is primarily dictated by the single-nucleotide polymorphism (SNP) rs17822931 (538G > A) in the ABCC11 gene (7–9). The wild-type allele encodes a functional ATP-binding cassette transporter (MRP8), an apical efflux pump located on the cellular membrane of apocrine sweat glands. This transporter actively secretes a plethora of initially odorless precursors—including L-glutamine conjugates and apolipoprotein D—into the glandular lumen. Histologically, patients with AO exhibit significant hyperplasia and hypertrophy of these apocrine glands, which densely cluster at the deep dermal-subcutaneous interface and are highly responsive to androgenic stimulation during and after puberty (10).

At the microbiological level, the sterile, organic-rich precursors secreted by the apocrine glands are subsequently catalyzed by the specialized axillary microbiome. High-resolution metagenomic studies have pinpointed specific bacterial taxa, predominantly Corynebacterium species and Staphylococcus hominis, as the primary biochemical culprits (11). These bacteria utilize unique enzymes to cleave the odorless precursors into highly volatile organic compounds (VOCs). These VOCs—primarily comprising volatile short-chain fatty acids like (E)-3-methyl-2-hexenoic acid (E3M2H) and volatile thioalcohols are the direct molecular source of the clinical malodor (12, 13).

### 1.2 Comprehensive landscape of therapeutic modalities

Because genetic editing of the ABCC11 allele remains clinically unfeasible (14), effectively blocking or extirpating the apocrine secretion pathway serves as the only viable therapeutic strategy. Driven by the pressing psychosocial needs of patients, the therapeutic armamentarium for axillary osmidrosis has evolved into a highly diverse spectrum. These modalities can be systematically classified into non-surgical treatments, energy-based devices, and surgical interventions, as comprehensively summarized in Table 1.

**Table 1:** Comprehensive summary and evolution of therapeutic modalities for axillary osmidrosis.

Category	Specific modalities	Primary mechanism	Clinical advantages & major limitations	Ref.
Non-surgical	Botulinum toxin type A (BTX-A) injections	Sympathetic denervation of local sweat glands	Provides reliable temporary odor reduction; requires repeated injections.	(15)
	Sclerosing agents (e.g., ethanol, polidocanol)	Chemical ablation and subsequent fibrotic tissue atrophy	Minimally invasive with low cost; carries a risk of skin necrosis and inconsistent long-term efficacy.	(16, 17)
Energy-based	Microwave thermolysis (e.g., miraDry)	Targeted deep dermal thermal coagulation	Scarless with quick recovery; high cost and potential incomplete ablation in severe Grade III cases.	(6)
	Nd:YAG laser, ultrasound lipoplasty	Thermal destruction or cavitation of apocrine glands	Minimal downtime; exhibits higher recurrence rates than definitive radical surgery.	(18, 19)
Surgical	Endoscopic shavers, Versajet hydro-surgery	Mechanical shaving or fluid jet debridement	Reduced incision size; the blind nature often leads to incomplete clearance of deep glands.	(20, 21)

First-line conservative therapies typically involve non-surgical and pharmacological interventions. Topical agents and oral anticholinergics provide temporary relief by minimizing bacterial colonization or suppressing overall sweat production. For targeted regional intervention, local injectables are widely utilized. Subcutaneous administration of botulinum toxin type A temporarily inhibits apocrine secretion via sympathetic denervation, providing reliable short-term relief (15). Furthermore, percutaneous injections of various sclerosing agents, notably absolute ethanol (16) and polidocanol (17), aim to induce targeted chemical ablation and subsequent fibrotic atrophy of the glandular structures.

Advancements in bioengineering have introduced minimally invasive energy-based devices (EBDs) designed to thermally destroy apocrine tissues. This category encompasses microwave thermolysis, subcutaneous pulsed Nd:YAG laser ablation, and micro-incision ultrasound-assisted lipoplasty (18, 19, 22, 23). While these technologies successfully circumvent severe surgical scarring, their finite energy penetration depth often fails to completely eradicate the hypertrophic deep-dermal glands characteristic of severe osmidrosis cases. Consequently, network meta-analyses and long-term comparative studies have demonstrated that EBDs are frequently associated with higher recurrence rates compared with traditional open surgery (6).

Physical surgical extirpation remains the ultimate curative approach, and the surgical paradigm has undergone a remarkable evolution to balance extensive clearance with aesthetic preservation. Early aggressive methods, such as wide fusiform skin excision or direct-vision flap eversion, guaranteed clearance but routinely resulted in catastrophic cicatricial contractures. This severe morbidity prompted the development of minimally invasive single modalities, which include endoscopic shaver excision (20), Versajet hydro-surgery (21), and pure negative-pressure liposuction. However, single blind methods often yielded unacceptable recurrence rates. To mitigate this, surgeons began synergizing techniques, leading to dual combinations such as tumescent liposuction combined with blind subcutaneous pruning or scrape-and-suction techniques. For highly recalcitrant cases, complex multimodal operations are increasingly employed, integrating suction, curettage, and direct-vision flap trimming within a single comprehensive procedure to optimize glandular clearance.

### 1.3 The surgical paradox and study objective

Despite this extensive array of therapeutic options, reconstructive surgeons persistently navigate a classic anatomical dilemma characterized as the subdermal vascular paradox. Aggressive open techniques provide excellent visualization and clearance but indiscriminately amputate the delicate subdermal vascular plexus, leading to devastating flap necrosis and restrictive scarring. Conversely, purely blind minimally invasive techniques perfectly preserve vascularity but frequently fail to detach terminal apocrine glands tethered to the deep dermis, culminating in frustrating recurrence rates.

To effectively mitigate this historical paradox, our institution has optimized a specific hybrid protocol utilizing modified small-incision tumescent suction-defatting followed immediately by direct-vision micro-trimming. This study aims to comprehensively validate the micro-surgical biomechanics and comparative clinical outcomes of this optimized technique against traditional resection in an 86-patient retrospective cohort.

## 2 Methods

### 2.1 Study design and patient cohort

We conducted a retrospective comparative study adhering to the Declaration of Helsinki with Institutional Review Board approval. Written informed consent was obtained. To assemble the cohort, we reviewed the medical records of consecutive patients evaluated for surgical treatment of bilateral AO at our institution between January 2022 and September 2025. Inclusion criteria were: (1) primary bilateral AO presenting with distinct axillary malodor (Park Grade II or III); and (2) age ≥ 18 years. Exclusion criteria were: (1) previous axillary surgeries or energy-based treatments; (2) severe systemic or bleeding disorders; and (3) active local infections. Initially, 94 consecutive eligible patients were identified. Eight patients were excluded during the study period (3 due to incomplete medical records and 5 lost to follow-up), yielding a final cohort of 86 patients.

Patients were allocated to the observation group (*n* = 43, modified technique) or the control group (*n* = 43, traditional small-incision resection). Treatment allocation was determined through shared decision-making and patient preference following detailed preoperative counseling. Because this allocation was non-randomized, we explicitly acknowledge the inherent potential for selection bias. As shown in Table 2, baseline demographic and clinical characteristics were statistically comparable between the two cohorts (*P* > 0.05).

**Table 2:** Baseline characteristics and clinical outcomes at 6 months postoperatively.

Variables	The modified technique (*n* = 43)	Traditional resection (*n* = 43)	*P*-value
Baseline characteristics			
Age (years), mean ± SD	24.5 ± 3.8	25.1 ± 4.2	0.489*
Gender (Male/Female)	14/29	16/27	0.821**
Park grade (II/III)	18/25	20/23	0.828**
Perioperative outcomes			
Intraoperative bleeding (mL), mean ± SD	8.5 ± 2.0	22.4 ± 4.5	<0.001*
Wound healing time (days), mean ± SD	10.2 ± 1.5	13.5 ± 2.1	<0.001*
Overall complications, *n* (%)	2 (4.65%)	9 (20.93%)	0.049**
– Minor bruising (*n*)	2	2	
–Subcutaneous indurations (*n*)	0	4	
– Delayed healing (*n*)	0	3	
Primary outcomes (6-Month Follow-up)			
Clinical efficacy, *n* (%)			1.000***
Cured	38 (88.37%)	37 (86.05%)	
Markedly effective	4 (9.30%)	4 (9.30%)	
Treatment failure	1 (2.33%)	2 (4.65%)	
Patient satisfaction, *n* (%)			0.021****
Fully satisfied (Overall Satisfaction)	40 (93.02%)	31 (72.09%)	
Generally satisfied	2 (4.65%)	8 (18.60%)	
Unsatisfied	1 (2.33%)	4 (9.30%)	

SD = standard deviation.

*Calculated using the independent Student's t-test for continuous variables.

** Calculated using Fisher's exact test for categorical variables.

*** Fisher's exact test comparing overall effective rate [(Cured + Markedly effective) vs. Treatment failure].

**** Fisher's exact test comparing overall satisfaction rate [Fully satisfied vs. (Generally satisfied + Unsatisfied)]. *P* < 0.05 is considered statistically significant.

### 2.2 Surgical techniques and Standard Operating Procedure (SOP)

#### 2.2.1 The modified technique

To explicitly elucidate the tissue-preserving biomechanics, we illustrate the procedure utilizing a representative 24-year-old female patient with severe, refractory bilateral AO (Park Grade III, carrying the wet earwax phenotype) (Figure 1).

**Figure 1:**
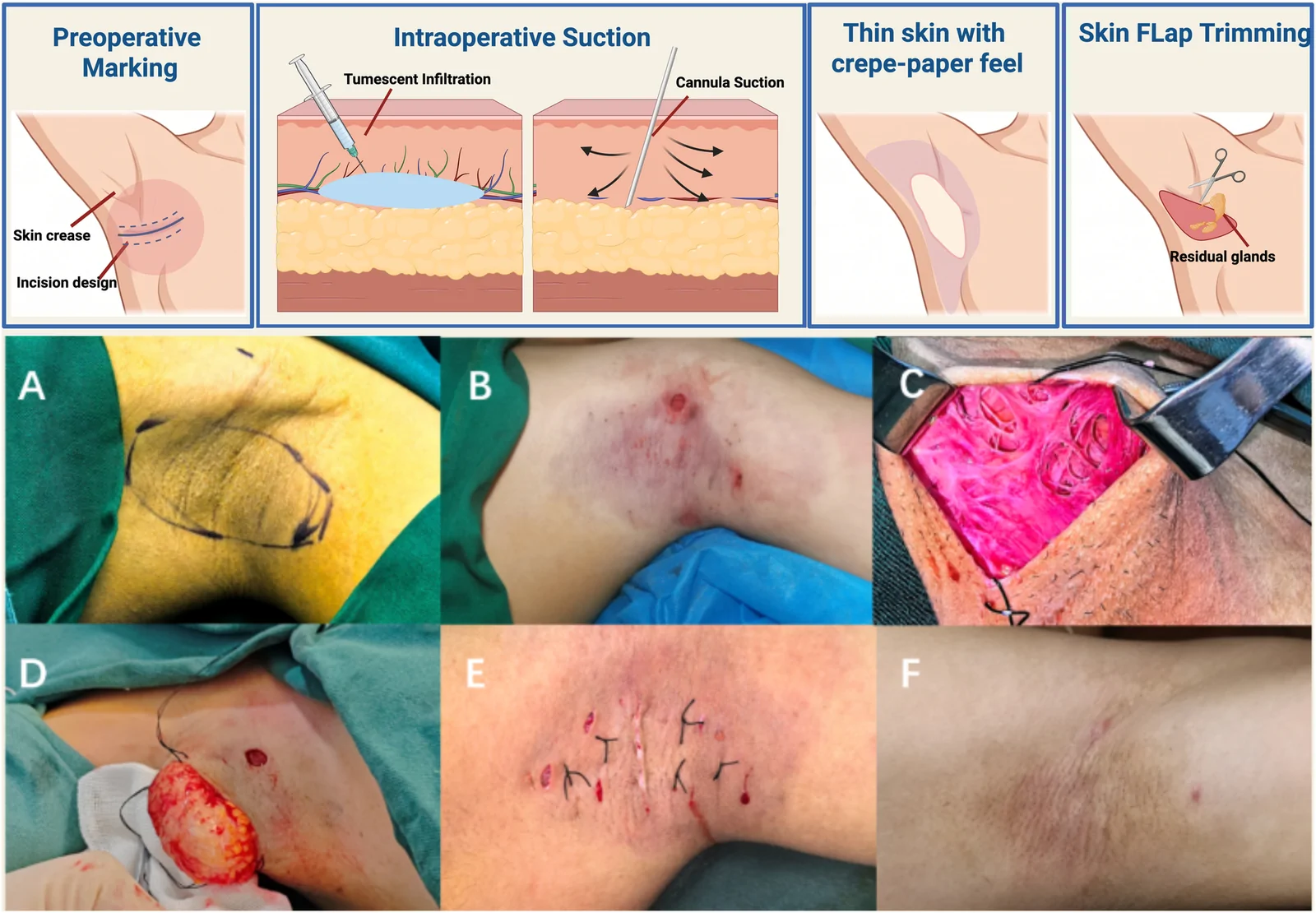
Surgical steps of the modified minimal incision curettage-scissors-suction technique. **(A)** Preoperative marking; **(B)** Intraoperative suction; **(C)** Formation of distinct tissue planes after suction; **(D)** Eversion of the skin flap to trim apocrine glands; **(E)** Immediate postoperative result; **(F)** 6 months postoperative.

##### Phase I: tumescent hydro-dissection

A micro-incision (1.0–1.5 cm) was designed along the central axillary crease. Approximately 100 mL of tumescent anesthetic solution was aggressively infiltrated, acting as a “liquid scalpel” to naturally dissect the apocrine-bearing adipose layer from deeper tissues while inducing profound vasoconstriction.

##### Phase II: atraumatic defatting via blunt avulsion

A 1.8-mm blunt-tipped liposuction cannula was introduced. Through fan-shaped reciprocating motions, negative pressure selectively aspirated loose adipose tissue, hair follicles, and easily detachable glands. Crucially, the blunt cannula respects tissue biomechanics, bypassing and preserving the tough collagenous neurovascular bundles.

##### Phase III: direct-vision micro-trimming

To minimize surgical blind spots, we gently everted the micro-incision. With the visually obstructive adipose tissue completely aspirated, residual yellowish apocrine glands tethered to the deep dermis were starkly exposed and meticulously trimmed using ophthalmic scissors under direct vision. The incision was closed with 5-0 sutures, followed by a compressive bandage.

#### 2.2.2 Traditional small-incision resection

In the control group, a 3–4 cm axillary incision was made. Following sharp subcutaneous dissection to raise a full-thickness skin flap, the glands and partial deep dermis were extensively excised using tissue scissors under direct vision without prior suction-defatting (Figure 2).

**Figure 2:**
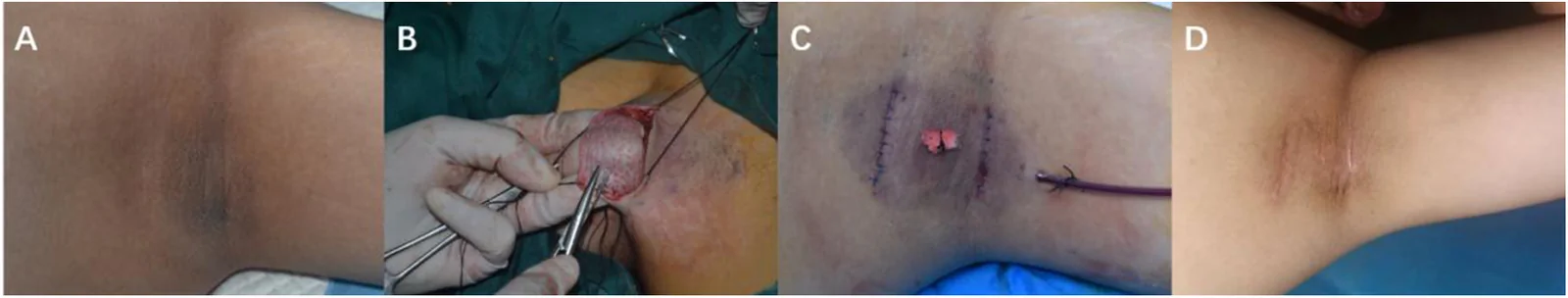
Surgical steps of the traditional minimal incision technique. **(A)** Preoperative appearance; **(B)** Eversion of the skin flap to trim apocrine glands; **(C)** Immediate postoperative result; **(D)** 6 months postoperative.

### 2.3 Postoperative evaluation and statistical analysis

Clinical efficacy was assessed at 6 months postoperatively using Park's criteria (Cured, Markedly effective, Treatment failure). Subjective patient satisfaction (categorized as “Fully satisfied”, “Generally satisfied”, and “Unsatisfied”) was recorded. Data were analyzed using SPSS 22.0. Continuous variables were compared using independent t-tests. Categorical variables were analyzed using Fisher's exact test due to small sample sizes in subcategories (*P* < 0.05 considered significant). “Overall patient satisfaction” was strictly defined as the proportion of patients reporting being “Fully satisfied”.

## 3 Results

### 3.1 Comparable clinical efficacy

At 6 months postoperatively, both modalities exhibited excellent odor control, effectively reducing the anatomical substrate necessary for bacterial VOC production. The total effective rate was 97.67% (42/43) in the observation group and 95.35% (41/43) in the control group (Fisher's exact test, *P* = 1.000). This indicates no statistically significant difference in effectiveness, demonstrating that the micro-incision hybrid technique achieves clinical efficacy comparable to open surgery.

### 3.2 Substantial reduction in morbidity and superior satisfaction

As detailed in Table 2, patients in the modified group experienced significantly shorter wound healing times (10.2 ± 1.5 days vs. 13.5 ± 2.1 days, *P* < 0.001) and minimal intraoperative bleeding (8.5 ± 2.0 mL vs. 22.4 ± 4.5 mL, *P* < 0.001). Complications in the modified group were significantly lower (4.65% vs. 20.93%, *P* = 0.049) and limited to minor bruising. Conversely, the traditional group experienced higher incidences of palpable subcutaneous indurations and delayed healing. Consequently, overall patient satisfaction was significantly higher in the observation group (93.02%) compared to the control group (72.09%) (Fisher's exact test, *P* = 0.021, Table 2).

## 4 Discussion

### 4.1 Translating basic science into surgical rationale

The integration of foundational biomolecular science dictates the strict necessity of surgical intervention in AO. As elucidated in the pathogenesis, the malodor is a downstream product of the ABCC11-mediated precursor efflux and subsequent specific microbiological enzymatic cleavage (7, 12). Because current medical science cannot permanently alter the genetic code of the MRP8 pump, and topical microbiome modifications (e.g., antibacterials) are strictly temporary, the extensive physical removal of the deep-dermal apocrine glands serves as the crucial therapeutic step. Our retrospective cohort study indicates that combining tumescent suction-curettage with direct-vision trimming successfully achieves this extensive removal while mitigating the historical surgical dilemma.

### 4.2 Mitigating the subdermal vascular dilemma

Chinese researchers have extensively explored combining small incisions with negative pressure. Early hybrid methods combined subcutaneous trimming with suction curettage. However, many early procedures performed the trimming phase blindly. Blind manual trimming significantly increases the risk of hematoma and leaves behind residual glands compared to direct-vision trimming (24). A recent 2025 comparative study by Liu et al. confirmed that pure liposuction-curettage (without trimming) yielded a significantly lower efficacy rate (87.8%) compared to visually guided excision (98.9%) (25). Furthermore, other contemporary approaches such as retrograde *en bloc* apocrine glandectomy also emphasize the strict necessity of direct visual clearance to prevent recurrence (26). As blind suction inevitably fails to detach terminal glands firmly embedded in the dermis. Furthermore, multi-modal approaches utilizing electrocautery or patented rubbing devices add complexity, cost, and potential thermal injury risks (27).

While our technique builds upon these established hybrid methods, it is important to clarify that the present study does not represent an entirely novel surgical concept, but rather a systematic optimization of existing surgical principles. In conventional suction-curettage procedures, negative pressure is often aggressively utilized to blindly avulse glands, which increases the risk of indiscriminate damage to the subdermal vascular plexus and leaves residual tethered glands. The specific incremental novelty of our protocol lies in redefining the role and sequence of the suction phase. We strictly restrict the blunt cannula to an atraumatic defatting process. Rather than relying on suction for extensive gland removal, it serves exclusively to clear the visually obstructive adipose matrix while preserving the robust collagenous neurovascular septa. Once defatted, the surgical field is transformed into a clear 2D plane. This uncoupling of the defatting and trimming phases aims to maximize targeted glandular clearance while explicitly prioritizing vascular preservation.

Our modified approach intelligently streamlines these biomechanical principles. The initial negative-pressure suction functions strictly as an atraumatic defatting phase. Rather than relying on suction to completely cure the disease, it serves to clear the visually obstructive adipose matrix while safely preserving the tough subdermal vascular septa. Once defatted, the surgical field is transformed into a clear 2D canvas. By employing a gentle eversion technique through a mere 1.2 cm incision—building upon the “pinch and turn-over” concept advocated by Lee (28),which surgeon can visually inspect the entire deep dermis. This final direct-vision micro-trimming acts as an anatomical fail-safe. It facilitates a 97.67% effective rate by surgically excising the visualized ABCC11-expressing apocrine glands, substantially depriving the axillary microbiome of the precursors needed to generate E3M2H malodor, while aiming to optimally preserve the subdermal vascular plexus.

Moreover, compared to techniques requiring bilateral double-incision liposuction, our protocol utilizes standard liposuction cannulas and a single micro-incision, making it highly accessible. Restricting the incision to 1.5 cm drastically reduces radial tension, suppressing fibroblast overproliferation and yielding highly inconspicuous scars. However, this study is subject to several limitations, including its retrospective, single-center design, a relatively moderate sample size, and the inherent selection bias introduced by the patient-preference-based allocation. Furthermore, vascular preservation and glandular clearance were assessed clinically rather than via objective histological or microvascular perfusion measurements. Moreover, the 6-month follow-up period is relatively short and may not capture late recurrences. Prospective, multi-center trials incorporating objective molecular quantification of axillary VOCs and longer observation periods are warranted to further corroborate long-term efficacy.

## 5 Conclusion

The etiology of axillary osmidrosis is deeply rooted in the genetic-microbiome axis, making physical extirpation of apocrine glands a highly reliable treatment. By integrating atraumatic negative-pressure tumescent suction-defatting with targeted direct-vision trimming, this modified paradigm effectively addresses the subdermal vascular paradox inherent in traditional AO surgery. It achieves comparable efficacy to traditional open resection while accelerating recovery, minimizing complications, and providing favorable aesthetic outcomes, establishing a highly reproducible and scientifically sound surgical option.

## Data Availability

The original contributions presented in the study are included in the article/Supplementary Material, further inquiries can be directed to the corresponding author.
